# A High-Q Resonant Pressure Microsensor with Through-Glass Electrical Interconnections Based on Wafer-Level MEMS Vacuum Packaging

**DOI:** 10.3390/s141224244

**Published:** 2014-12-16

**Authors:** Zhenyu Luo, Deyong Chen, Junbo Wang, Yinan Li, Jian Chen

**Affiliations:** 1 State Key Laboratory of Transducer Technology, Institute of Electronics, Chinese Academy of Sciences, Beijing 100190, China; E-Mails: oreilzy@gmail.com (Z.L.); yzngb@163.com (Y.L.); chenjian@mail.ie.ac.cn (J.C.); 2 University of Chinese Academy of Sciences, Beijing 100190, China

**Keywords:** resonant, pressure sensor, through-glass via, anodic bonding, Q-factor, vacuum packaging

## Abstract

This paper presents a high-Q resonant pressure microsensor with through-glass electrical interconnections based on wafer-level MEMS vacuum packaging. An approach to maintaining high-vacuum conditions by integrating the MEMS fabrication process with getter material preparation is presented in this paper. In this device, the pressure under measurement causes a deflection of a pressure-sensitive silicon square diaphragm, which is further translated to stress build up in “H” type doubly-clamped micro resonant beams, leading to a resonance frequency shift. The device geometries were optimized using FEM simulation and a 4-inch SOI wafer was used for device fabrication, which required only three photolithographic steps. In the device fabrication, a non-evaporable metal thin film as the getter material was sputtered on a Pyrex 7740 glass wafer, which was then anodically bonded to the patterned SOI wafer for vacuum packaging. Through-glass via holes predefined in the glass wafer functioned as the electrical interconnections between the patterned SOI wafer and the surrounding electrical components. Experimental results recorded that the Q-factor of the resonant beam was beyond 22,000, with a differential sensitivity of 89.86 Hz/kPa, a device resolution of 10 Pa and a nonlinearity of 0.02% F.S with the pressure varying from 50 kPa to 100 kPa. In addition, the temperature drift coefficient was less than −0.01% F.S/°C in the range of −40 °C to 70 °C, the long-term stability error was quantified as 0.01% F.S over a 5-month period and the accuracy of the microsensor was better than 0.01% F.S.

## 1. Introduction

High-accuracy barometric pressure microsensors have been the subject of extensive research due to their applications in the fields of aerospace exploration, atmospheric pressure monitoring, *etc.* [1]. Depending on the detection mechanisms, these microsensors can be classified into capacitive pressure sensors [2,3], piezoelectric pressure sensors [4,5], piezoresistive pressure sensors [6,7] and resonant pressure sensors [8]. Compared to the non-resonant pressure sensors, resonant pressure sensors present the advantage of “quasi-digital” output, which allows easy coupling to digital electronics and thereby results in high resolution and reliability. Additionally, resonant pressure sensors have excellent long-term stability since the resonance frequency is not dependent on unstable or drifting electrical signals, but rather on the mechanical properties of the structure [9,10].

The mechanical quality factor of a microresonator deteriorates as the environmental pressure increases due to the air damping effect [11,12]. To achieve high performance, resonators are commonly isolated from the environment by vacuum packaging [13,14]. However, for MEMS-based pressure sensors, the vacuum packaging is different from the conventional packaging counterparts based on ceramic or metal hermetic sealing, which can lead to compromise of the MEMS devices during the manufacturing process due to their movable and fragile structures. Thus, wafer-level bonding which can protect the MEMS devices from subsequent processes is preferred to realize vacuum packaging [15].

Wafer-level vacuum packaging has the advantages of small size, low cost and compatibility with micro-fabrication processes. Bonding the cap wafer with the device substrate is the main sealing process for MEMS devices. The micro-cap can offer robust protection, protecting the fragile mechanical parts from possible impact and destruction by after-processes [16,17].

A variety of bonding techniques have been proposed for wafer-level vacuum packaging, such as intermediate layer bonding, silicon–silicon fusion bonding, and silicon–glass anodic bonding [18]. Among these approaches, the electrical interconnections between the bonding micro-cap and the device substrate pose several challenges for vacuum packaging. They use valuable die area, are often the source of failure or leakage, and complicate cavity sealing due to the added process steps [19].

**Figure 1.**
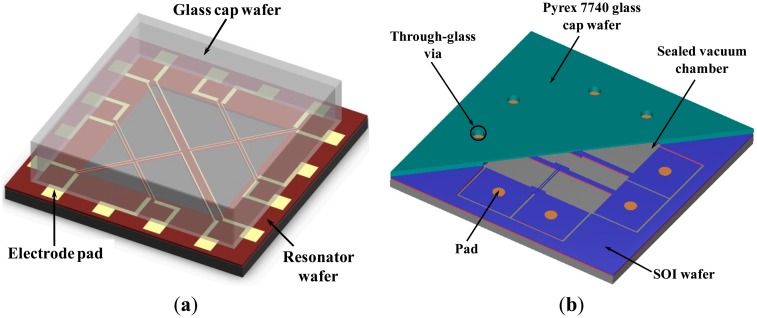
The basic structure diagrams of two wafer-level vacuum packaging designs. (**a**) Previous work based on low-temperature adhesive bonding with patterned electrodes across the bonding interface; (**b**) New design presented in this paper with SOI-glass anodic bonding and through-glass via holes, which provide electrical interconnections with the outside.

Our previous work [13,20] utilized a non-photosensitive BCB-based low-temperature adhesive bonding technique to realize vacuum encapsulation. This organic glue-based adhesive bonding [21,22] has the advantages of relatively low bonding temperature, good design flexibility and low commercial cost. In addition, this approach can facilitate electrical connections with the outside by patterning electrodes across the bonding interface (Figure 1a). However, limited by the physical properties of organic materials, the poor long-term vacuum tightness is its fatal defect.

To address these issues, through-glass vias [23,24] and silicon-to-glass anodic bonding technologies were utilized in this study to fabricate a resonant pressure sensor consisting of an SOI wafer and a Pyrex 7740 glass wafer [25,26]. Presently, the SOI substrate has been extensively employed in MEMS devices because of its simple yet reliable fabrication steps, higher yield and robust structures. The fabrication procedures for the proposed sensor were based on simplified SOI-MEMS fabrication processes requesting only three photolithographic steps.

Compared to previous designs, this new device has several advantages:

(I) The top single-crystal silicon layer of the SOI wafer was used to fabricate the resonators due to its superb mechanical properties and high intrinsic quality factors. In addition, the proposed pressure microsensor adopts a differential structure based on two resonators with a comparable temperature dependency to suppress the temperature effect.
(II) A wafer-level vacuum packaging using the silicon-to-glass anodic bonding technique was utilized to form a sealed vacuum chamber, guaranteeing long-term vacuum tightness. The anodic bonding technique [18] possesses the advantages of the relatively low required temperature (350 °C–450 °C) and high bonding strength and hermeticity. In this paper, a Pyrex 7740 glass wafer used as the cap wafer was anodic bonded to the patterned SOI wafer for vacuum packaging.
(III) A through-glass via (TGV) technology was used. After anodic bonding was complete, the aluminum film was sputtered into the via holes by a shadow-mask technique to form the contact pads for electrical interconnections [26]. This approach enabled the resonators to be electrically connected to outside by wire bonding through the front side of the assembled wafers.
(IV) A non-evaporable metal thin film as the getter material was embedded into the concave of the Pyrex 7740 glass cap to help maintain the high vacuum condition and produce high quality factors of resonators. The use of the getter film for wafer-level vacuum packaging was described elsewhere [27], which has two functions. First, the metal film acts as a diffusion barrier to gas atoms. Second, getter materials such as titanium have the ability to absorb common gases when activated at certain temperatures. Thus the getter material reduces the trapped gases inside the cavity as well as the outgassing generated during the anodic bonding process.

As shown in Figure 1b, through-glass vias were realized using laser drilling into a Pyrex 7740 glass wafer. Then, hermetic sealing was achieved by anodic bonding the processed glass cap wafer with a patterned SOI wafer. The vias provide electrical signal paths to the pressure device.

## 2. Device Design

**Figure 2.**
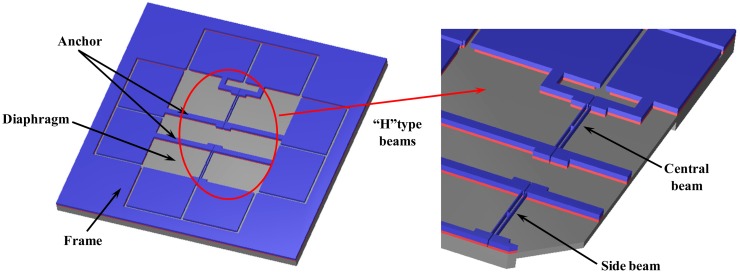
The schematic diagrams of the differential pressure sensor fabricated on a SOI wafer include a pressure-sensitive silicon square diaphragm and two “H” type doubly-clamped resonant beams. Pressure under measurement causes a deflection of the diaphragm, which is translated to an axial tensile stress in the central beam and an axial compressive stress in the side beam, leading to resonant frequency shifts towards opposite directions enabling a differential output.

The schematic diagram of the differential resonant pressure sensor fabricated on a SOI wafer is shown in Figure 2. The resonant elements consist of two “H” type doubly-clamped beams suspended on a pressure-sensitive silicon square diaphragm. The single-crystal silicon “H” type beams used as resonators work in a lateral mode, which are actuated and detected electromagnetically. The two beams named “central beam” (located in the center of the diaphragm) and “side beam” (located near the border of the diaphragm) have almost identical dimensions and thus comparable resonant frequencies at zero pressure loads [20,26]. In this device, pressure under measurement causes a deflection of the diaphragm, which is further translated to an axial tensile stress build up in the central beam while an axial compressive stress in the side beam, leading to resonant frequency shifts respectively towards opposite directions, enabling a differential output.

Like many sensors, however, the resonant barometric pressure sensor is still susceptible to the temperature variation of the ambient air. To address this issue, this design is featured with a differential signal output of the two resonant beams. In response to the temperature variation, the resonant frequencies of the two resonant beams drift in the same direction. The corresponding frequency drift is thus partially suppressed based on the differential setup. Other sources of errors such as gas density or humidity variation of the ambient air are also be nullified to a negligible degree by the high vacuum hermetic sealing condition, enhancing device stability and sensitivity.

## 3. FEM Simulation

Finite element modeling was used in the design and optimization of the resonant pressure micro- sensor to deal with two concerns: mode interference and two-beam sensitivity mismatch. Mode interference or the frequency overlap of two nearby resonant modes can drive the resonant beams to vibrate in undesirable modes, leading to mode crosstalk and energy loss with compromised quality factor. The sensitivity mismatch between the two resonant beams can lead to compromised performance in the differential resonant frequency output.

The structure of the pressure sensor was simulated using the ANSYS finite-element package which was initially defined using the solid module. The key dimensions were parameterized, enabling the rapid simulation of design modifications and the high-efficiency optimization routine. In this study, static simulations were used to calculate the stress distribution along the resonant micro beams as a function of pressure, with the purpose of optimizing the relative positions of the two resonant beams to address the issue of sensitivity mismatch. Then, mode simulations were used to locate the optimum vibration mode, which is capable of transferring stress of resonant beams into the intrinsic resonant frequency shift [26].

More specifically, static simulations based on ANSYS multi-physics Packaging were conducted as follows with the detailed material properties shown in Table 1. The element type “Solid 10-node 92” was used to mesh the prototype of the micro sensor for both static structural and mode analysis. The boundary area of the pressure sensitive diaphragm was completely restrained to prevent unconstrained movements. A series of pressure values were applied on the backside of the diaphragm followed by mode simulations to extract all the intrinsic modes within the frequency range from 0 to 100 kHz to locate the desirable vibration mode. Details of the simulation results are summarized as follows.

**Figure 3.**
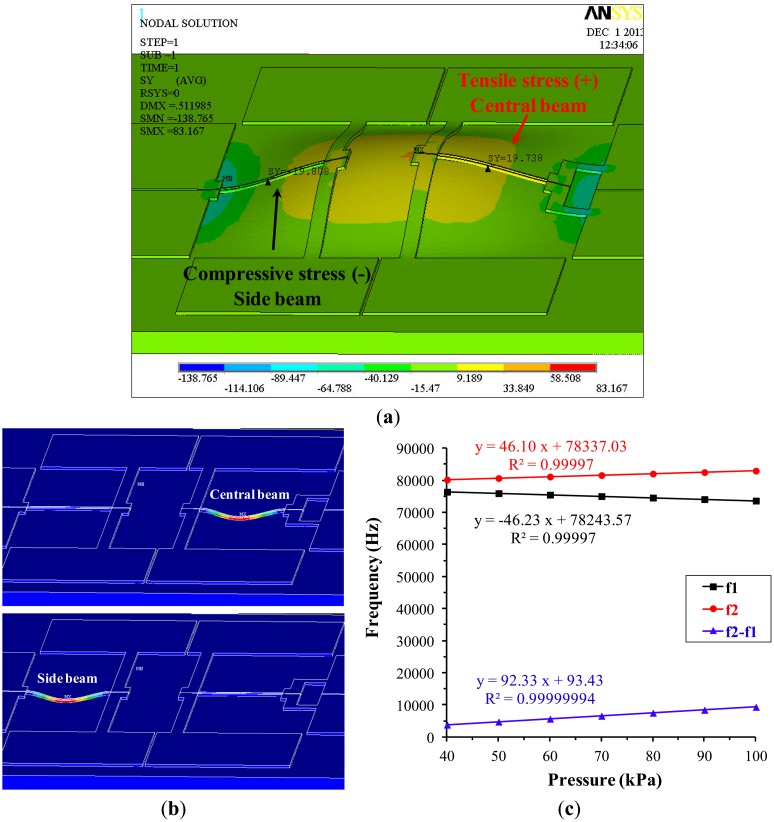
Simulation results. (**a**) The stress contour along the axial direction of the resonant beams under an applied pressure of 100 kPa. Positive stress (tensile) was induced in the central beam, while negative stress (compressive) was observed in the side beam; (**b**) Maximal displacements of the two resonant beams in the first order lateral vibration mode; (**c**) Simulated resonant frequencies of the two resonant beams as a function of applied pressure.

Figure 3a represents the stress contour along the axial direction of the beams under an applied pressure of 100 kPa. Positive stress (tensile) was induced in the central beam with a characterized value of 19.7 MPa, while negative stress (compressive) was observed in the side beam with a quantified value of −19.8 MPa. Based on the analysis, two resonant beams were under comparable stresses, guaranteeing identical sensitivities of resonant beams and enabling a differential output.

Figure 3b shows the optimum vibration mode for the resonant beams featured with lateral vibration within the wafer plane. Lateral vibrations are preferred in this study since its vibration cannot lead to the co-vibration of the sensitive diaphragm where the energy loss is minimized. Figure 3c shows the FEM simulation results of resonant frequencies of the side beam (f1) and the central beam (f2) as a function of applied pressure. The linear coefficients of both beams were quantified as 0.9999 and the linear correlation coefficient of the differential output (f2−f1) was quantified as 0.9999999. In addition, a twofold sensitivity of the differential output compared to the case of single beam was also noticed.

## 4. Device Fabrication

**Figure 4.**
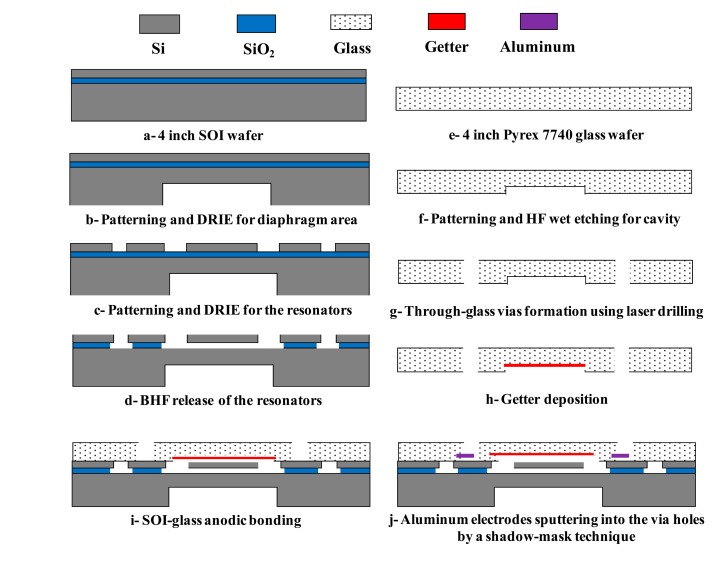
The fabrication procedure for the resonant pressure micro sensor, relying on SOI-MEMS fabrication processes.

Conventional MEMS bulk-silicon fabrication processes including deep reactive ion etching (DRIE), photolithography and sacrificial layer release were used to fabricate the proposed resonant pressure micro sensor starting from a standard 4 inch (101.6 mm) SOI wafer (40 + 2 + 300 (μm)). The fabrication process is described in Figure 4. Furthermore, through-glass vias were realized using laser drilling into a Pyrex 7740 glass wafer (500 μm).

The fabrication of the resonators was based on a 4 inch SOI wafer (Figure 4a–d). Only two photolithographic steps were needed to create the resonators. Initially, using a patterned positive photoresist as the mask, the handle layer was etched by DRIE to the depth of 120 μm to define the pressure-sensitive diaphragm (Figure 4b). Secondly, using patterned aluminum film and positive photoresist as the mask, the exposed device layer of the SOI wafer was etched by DRIE to a depth of 40 μm to define the resonant beams (Figure 4c). Then, the SOI wafer was immersed in a buffered hydrofluoric acid (BHF) solution, releasing the resonant beams by undercutting the insulation layer in a time-controlled manner (Figure 4d).

For the cap wafer, a Pyrex 7740 glass wafer (500 μm) was used. The fabrication process flow of the cap wafer is shown in Figure 4e–h. Based on deposition and patterning of a metallic mask, the cavity was etched using wet HF (Figure 4f). Then, after through-glass vias formation using laser drilling, the getter film was deposited inside the cavity (Figure 4h).

Next, silicon-to-glass anodic bonding was utilized to form a sealed vacuum chamber by anodic bonding the patterned SOI wafer with the Pyrex 7740 glass wafer (Figure 4i). Then, the Al electrodes were sputtered on the front side of the bonded SOI-glass wafer through the via holes by a shadow-mask technique to form electrical connections to the outside (Figure 4j). In the end, the fabricated wafer was diced into single sensor units.

**Figure 5.**
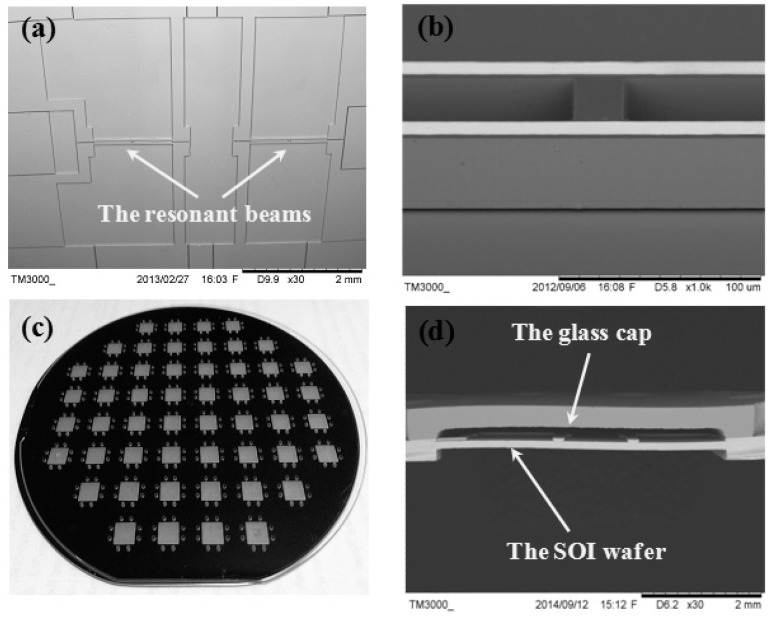
The SEM images of (**a**) the top view of the fabricated SOI wafer and (**b**) the suspended “H” type doubly-clamped beam; (**c**) The photograph of wafer-level anodic bonding between the Pyrex 7740 glass wafer with through-glass vias and the patterned SOI wafer; (**d**) The SEM image of the bonding interface of a sensor unit after wafer dicing.

The proposed resonant pressure micro sensor was successfully made by MEMS bulk micromachining. Figure 5a,b show the SEM images of the top view of the fabricated SOI wafer and the “H” type doubly-clamped beams, respectively [26]. As shown in Figure 5c, a Pyrex 7740 glass cap wafer with through-glass vias was anodic bonded to the patterned SOI wafer, forming a hermetic sealing for the resonators. Figure 5d shows the SEM image of the bonding interface of a sensor unit after wafer dicing.

## 5. Device Characterization

**Figure 6.**
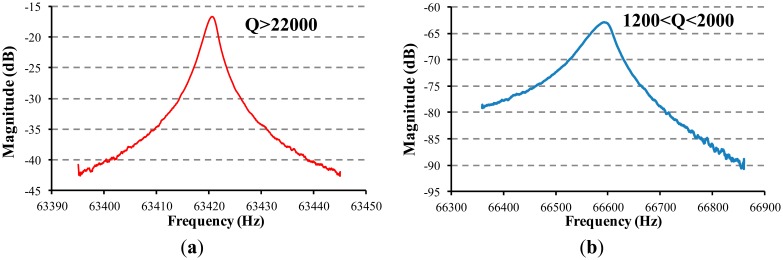
Open loop test to obtain the Q-factor of the resonant beam of the bonded SOI-glass wafer with (**a**) and without (**b**) the getter film deposited inside the cavity, which can easily demonstrate the getter material is effective.

The frequency response of the resonant beam of the proposed micro pressure sensor was measured by an E5061B Network Analyzer (Agilent, Santa Clara, CA, USA) in an open-loop scanning manner. The quantified Q-factor of the detection beam was higher than 22,000 (Figure 6a), which was stable for 5 months, confirming the reliability of the wafer-level vacuum packaging. In addition, Figure 6b shows that the Q-factor of the resonant beam of the bonded SOI-glass wafer without the getter film deposited inside the cavity was quantified as about 1800 [26]. This fact can easily demonstrate the getter material is effective.

**Figure 7.**
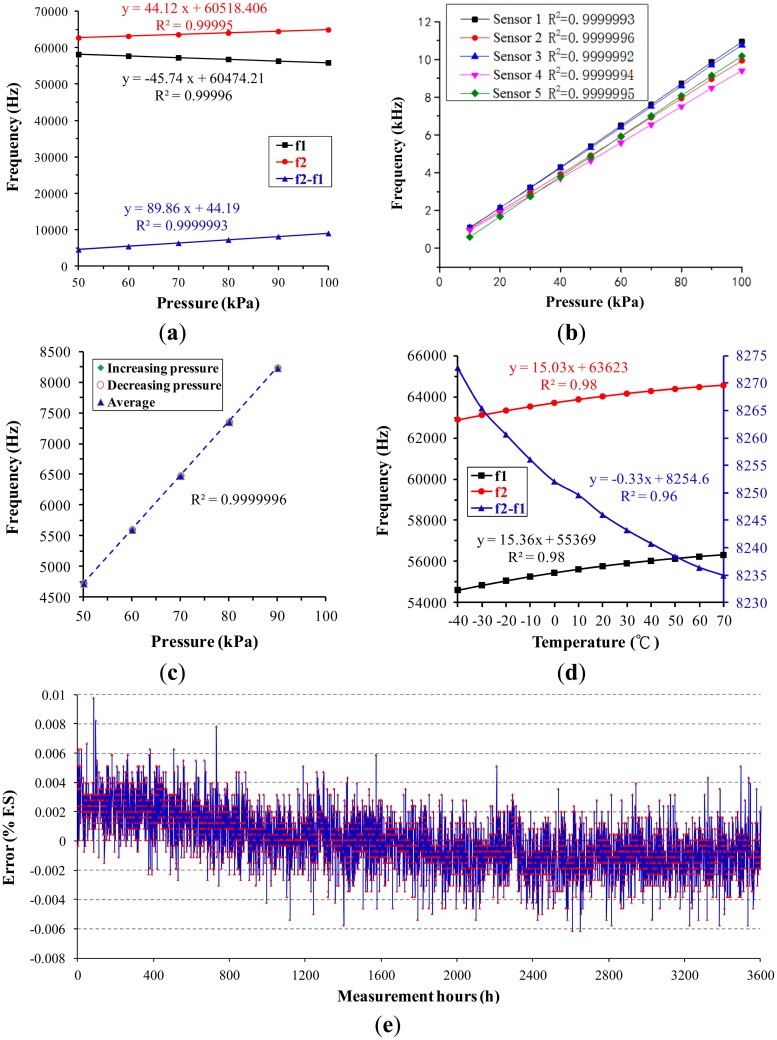
(**a**) The resonant frequencies of the side beam (f1) and the central beam (f2) and the differential frequency output (f2−f1) of a typical sensor versus applied pressure at 20 °C. Differential output with nearly balanced sensitivities of two beams improves the nonlinearity and sensitivity of the sensor; (**b**) The plot of the resonant frequency of 5 pressure sensors versus applied pressure, indicating a nonlinearity of 0.02% within the pressure range of 50 kPa to 100 kPa; (**c**) Quantified maximum hysteresis error of 0.03% F.S. and (**d**) resonant frequency shift as a function of the temperature under an applied pressure of 90 kPa in the range of −40 °C to 70 °C; (**e**) Zero-point long-term stability of the developed resonant pressure micro sensor, reporting a long-term stability error of 0.01% F.S over a 5-month period.

In the next step, a closed-loop self-oscillating circuit was used to further evaluate the device performance. Figure 7a records the resonant frequencies of the side beam (f1) and the central beam (f2) and the differential frequency output (f2−f1) as a function of applied pressure at 20 °C, quantifying the sensitivity of the two beams about 44.12 Hz/kPa (central beam) and 45.74 Hz/kPa (side beam), a differential sensitivity of 89.86 Hz/kPa and a resolution of about 10 Pa. The test result indicates that the output of the two beams was well balanced, and the differential output was shown to improve the nonlinearity and sensitivity of the sensor.

Figure 7b shows the plot of the resonant frequency of five pressure sensors versus applied pressure, indicating a nonlinearity of 0.02% within the pressure range of 50 kPa to 100 kPa. Figure 7c shows the maximum hysteresis error of 0.03% F.S. Figure 7d shows the resonant frequency shift as a function of the temperature under an applied pressure of 90 kPa in the range of −40 °C to 70 °C, which were quantified as about 15.03 Hz/°C (central beam) and 15.36 Hz/°C (side beam). Thus, the resonant frequency shift as a function of the temperature of the two beams varied identically, so that the differential output was suppressed to −0.33 Hz/°C, indicating the temperature coefficient of the sensor was less than −0.01% F.S/°C in the range of −40 °C to 70 °C. Figure 7e records zero-point long-term stability of the developed resonant pressure micro sensor, reporting a long-term stability error of 0.01% F.S over a 5-month period.

**Figure 8.**
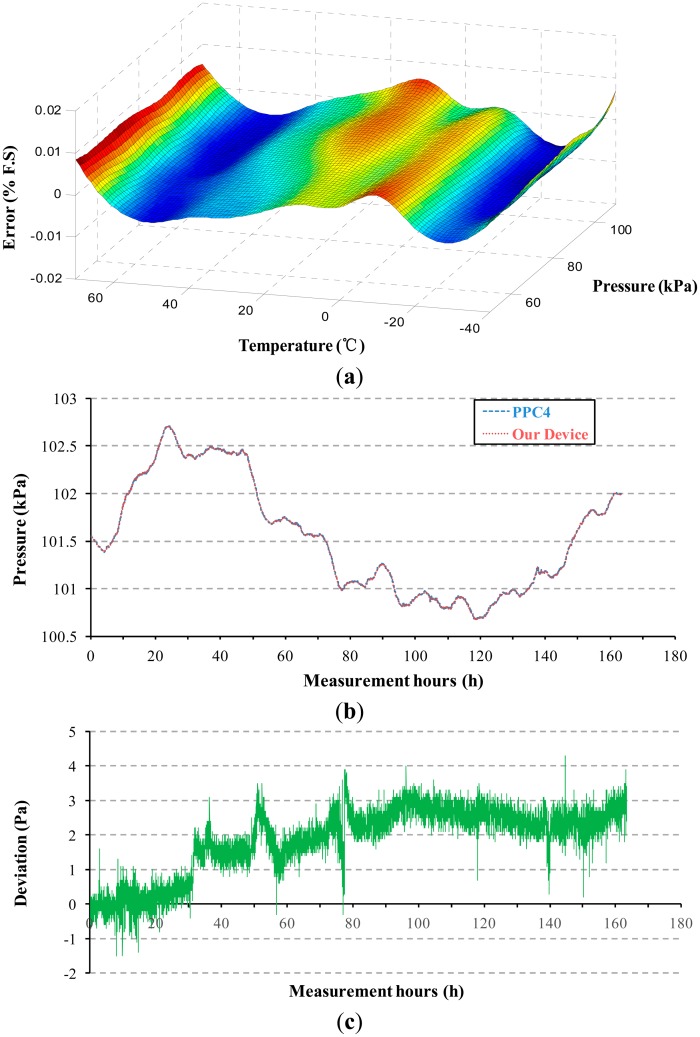
(**a**) Curve-fit accuracy, better than <0.01% of Full Scale; (**b**) Actual atmospheric pressure measurement of the device proposed in this study and PPC4 over a 7-day period, with pressure deviations shown in (**c**).

Equal importance to stability is the accuracy of the sensor device. The sensor device was packaged for operation from −40 °C to 70 °C and over this temperature range the frequency output of the device and temperature signal were combined by polynomial curve-fitting (Figure 8a) to create a performance of better than <0.01% F.S. In addition, Figure 8b,c show the results of the actual atmospheric pressure measurement of our sensor device compared with those obtained using a PPC4 Pressure Calibrator (Fluck, Everett, WA, USA) equipped with quartz reference pressure sensors over a 7-day period, indicating a maximum pressure deviation less than 5 Pa.

## 6. Conclusions

A high-Q resonant pressure microsensor with through-glass electrical interconnections based on wafer-level MEMS vacuum packaging was presented, which used the silicon-to-glass anodic bonding technique and the getter material preparation to maintain high-vacuum condition. A TGV technology was used to form electrical interconnections with the outside. The performance of the prototype sensor demonstrates the feasibility of the device design and MEMS fabrication process of the proposed sensor. Open-loop scanning measurements revealed the Q factor of the resonator was higher than 22,000. Furthermore, several performance measurements were conducted with closed-loop self-oscillating circuit. Over the pressure range of 50 kPa to 100 kPa, the resolution was about 10 Pa, and the nonlinearity was lower than 0.02%. In addition, the temperature drift coefficient was less than −0.01% F.S/°C in the range of −40 °C to 70 °C, and the long-term stability error was quantified as 0.01% F.S over a 5-month period and the accuracy of the micro sensor was better than 0.01% of full scale.

## Figures and Tables

**Table 1.** Parameter list used in FEM simulation.

**Items**	**Parameters**	**Values**	**Units**
Silicon	Young's Modulus	165	*GPa*
	Density	2.3	*g/cm^3^*
	Poisson's Ratio	0.24	

Beams	Length	1400	*μm*
	Width	20	*μm*
	Thickness	40	*μm*

Diaphragm	Length and Width	5100	*μm*
	Thickness	120	*μm*

Sensor Chip	Length and Width	10,200	*μm*
	Thickness	300	*μm*
